# Leiomyosarcoma of the skin with osteoclast-like giant cells: a case report

**DOI:** 10.1186/1752-1947-1-180

**Published:** 2007-12-14

**Authors:** Deba P Sarma, Eric E Santos, Bo Wang

**Affiliations:** 1 Department of Pathology, Creighton University Medical Center, Omaha, NE 68131, USA

## Abstract

**Introduction:**

Osteoclast-like giant cells have been noted in various malignant tumors, such as, carcinomas of pancreas and liver and leiomyosarcomas of non-cutaneous locations, such as, uterus and rectum. We were unable to find any reported case of a leiomyosarcoma of the skin where osteoclast-like giant cells were present in the tumor.

**Case presentation:**

We report a case of a 59-year-old woman with a cutaneous leiomyosarcoma associated with osteoclast-like giant cells arising from the subcutaneous artery of the leg. The nature of the giant cells is discussed in light of the findings from the immunostaining as well as survey of the literature.

**Conclusion:**

A rare case of cutaneous leiomyosarcoma with osteoclast-like giant cells is reported. The giant cells in the tumor appear to be reactive histiocytic cells.

## Introduction

Osteoclast-like giant cells have been noted in various malignant tumors, such as, carcinomas of pancreas and liver and leiomyosarcomas of non-cutaneous locations, such as, uterus and rectum. We were unable to find any reported case of leiomyosarcoma of the skin where osteoclast-like giant cells were present in the tumor. We are reporting such a case occurring in the leg of a 59-year-old woman and discussing the nature of the osteoclast-like giant cells in light of the results from the immunostaining as well as the survey of the literature.

## Case presentation

**Figure 1:**
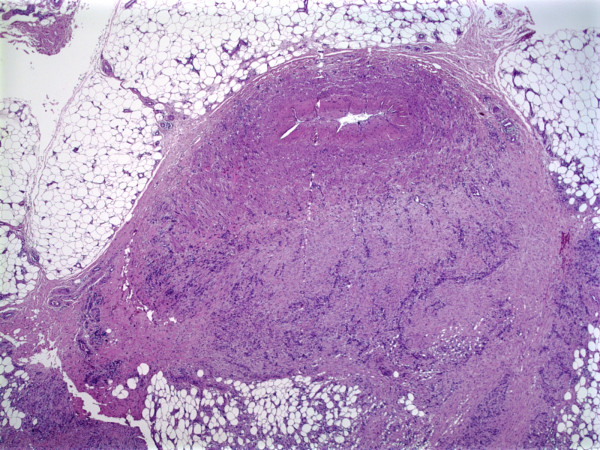
A subcutaneous spindle cell neoplasm arising from the muscular wall of an artery.

**Figure 2:**
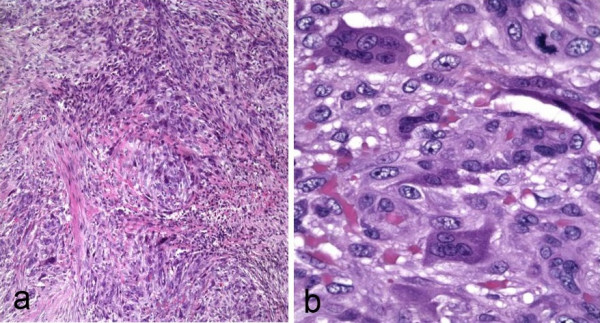
a. Intersecting fascicles of spindle cells with nuclear pleomorphism and dark giant cells. b. Osteoclast-like giant cells in the stroma between the spindle cells.

**Figure 3:**
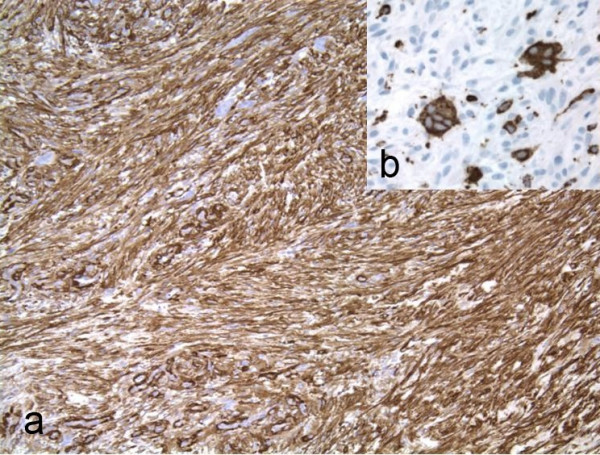
a. The neoplastic spindle cells are strongly positive for SMA (smooth muscle actin). b. The osteoclast-like giant cells are strongly positive for CD68.

A 59-year-old woman presented with a painless skin nodule on her left leg present for an unknown period of time. The patient's remaining medical history was unremarkable. An excisional biopsy of the leg nodule (Fig. 1) showed an infiltrating spindle cell neoplasm within the subcutaneous tissue, arising from the muscular wall of an artery. The tumor was composed of proliferating, interweaving fascicles of eosinophilic spindle cells with pleomorphic ovoid to cigar-shaped nuclei and occasional paranuclear vacuoles (Fig. 2a). The mitotic activity was brisk, ranging from 1 to more than 5 per 5 high-power fields. A striking finding in the tumor was the presence of scattered osteoclast-like giant cells with dark basophilic cytoplasm and multiple nuclei (Fig. 2b) in between the neoplastic spindle cells. The spindle cells were strongly immunoreactive to Vimentin and SMA (smooth muscle actin) (Fig. 3a) but non-reactive for CD68, CD31, cytokeratin AE1/3, S-100, and HHV-8. The osteoclast-like giant cells were negative for SMA but strongly positive for CD68 (Fig. 3b). The neoplasm was interpreted as a leiomyosarcoma with osteoclast-like giant cells. Approximately 25% of the neoplastic spindle cells were positive for the proliferative immunomarker, Ki 67.

## Discussion

Cutaneous leiomyosarcomas are classified as dermal, arising from the arrectores pilorum muscle, or subcutaneous, arising from the blood vessel wall [1]. Several histologic variants of the cutaneous leiomyosarcomas, such as, epithelioid [2] and granular cell type [3] have been reported. However, we were unable to find any reported case of a leiomyosarcoma of the skin where osteoclast-like giant cells were present in the tumor. Leiomyosarcomas with osteoclast-like giant cells arising in non-cutaneous locations, such as, uterus and rectum have been reported in the literature [4-6]. It can morphologically be confused with atypical fibroxanthoma (AFX)/cutaneous malignant fibrous histiocytoma (MFH), malignant melanoma, Kaposi sarcoma, spindle cell carcinoma, epithelioid angiosarcoma, and malignant peripheral nerve sheath tumor. The most difficult diagnostic dilemma for such a tumor is to distinguish it from an AFX/MFH. The histologic features and the immunohistochemical profile may be somewhat similar. However, the AFX/MFH typically occurs in the upper dermis of the sun-exposed skin, usually in the head and neck. It is not associated with pilar muscle or blood vessels. Immunohistochemically, the cells in AFX/MFH can be focally positive to smooth muscle actin, however, a strongly positive diffuse pattern is unusual. A subset of leiomyosarcomas is thought to arise from undifferentiated mesenchymal cells which may acquire smooth muscle features. However, the spindle cells and the giant cells of such a tumor are usually positive for CD68.

The tumor in our case clearly arises from muscle wall of an artery (Fig. 1) with histologic features of a leiomyosarcoma, including spindle cells with eosinophilic cytoplasm, oval or cigar-shaped nucleoli with paranucleolar vacuoles and immunologic feature of strongly SMA-positive tumor cells. On the other hand, the AFX/MFH is composed of fibroblastic cells with CD68 positivity. The predominant spindle cells of our tumor were negative for CD68. The only CD68-positive cells in the tumor were the osteoclast-like giant cells. We believe that our case represents a cutaneous leiomyosarcoma with reactive osteoclast-like giant cells based on the demonstration of origin from the arterial wall and histologic and immunologic evidence.

In addition to leiomyosarcoma, osteoclast-like giant cells have been noted in carcinomas of pancreas and liver [7]. The origin and nature of the osteoclast-like giant cells in various malignant tumors has remained controversial. However, most of the authors believe that the giant cells are of histiocytic origin and are reactive in nature. Features suggesting their benign nature include: bland appearance identical to osteoclasts in osteoclastoma, different immunostaining patterns from the malignant spindle cells, and no proliferating evidence, such as non-immunoreactivity to Ki67 [4-7].

The prognostic significance of osteoclast-like giant cells in cutaneous leiomyosarcoma is unknown. Dermal leiomyosarcomas are frequently recurrent, but almost never metastatic [8]. Conversely, subcutaneous leiomyosarcomas behave similar to those arising within deep soft tissue with frequent local recurrences and as much as 50% distant metastasis [9]. A complete excision with wide surgical margins should be the preferred treatment.

## Conclusion

A rare case of cutaneous leiomyosarcoma with osteoclast-like giant cells is reported. The giant cells in the tumor appear to be reactive histiocytic cells.

## Competing interests

The author(s) declare that they have no competing interests.

## Authors' contributions

BW reviewed the literature and drafted the manuscript. EES reviewed the immunostudies and revised the manuscript. DPS conceived, revised, and submitted the manuscript. All authors have read and approved the final manuscript.

## Consent

Written informed consent was obtained from the patient for publication of this case report.
